# Antimicrobial and Phytotoxic Activity of *Origanum heracleoticum* and *O. majorana* Essential Oils Growing in Cilento (Southern Italy)

**DOI:** 10.3390/molecules24142576

**Published:** 2019-07-16

**Authors:** Teresa Della Pepa, Hazem S. Elshafie, Raffaele Capasso, Vincenzo De Feo, Ippolito Camele, Filomena Nazzaro, Maria Rosa Scognamiglio, Lucia Caputo

**Affiliations:** 1 Department of Agricultural Sciences, University of Naples Federico II, via Università 100, 80055 Portici, Napoli, Italy; 2 School of Agricultural, Forestry, Food and Environmental Sciences, University of Basilicata, Viale dell’Ateneo Lucano 10, 85100 Potenza, Italy; 3 Department of Pharmacy, University of Salerno, Via Giovanni Paolo II 132, 84084 Fisciano, Salerno, Italy; 4 Institute of Food Science, CNR-ISA, Via Roma 64, 83100 Avellino, Italy

**Keywords:** *Origanum heracleoticum*, *Origanum majorana*, antimicrobial activity, phytotoxic activity, spore germination, minimum inhibitory concentration

## Abstract

There is a growing interest in a potential use of essential oils (EOs) as a replacement for traditional pesticides and herbicides. The aims of this study were to: (i) Identify the chemical composition of the two EOs derived from *Origanum heracleoticum* L. and *O. majorana* L., (ii) evaluate the in vitro antifungal activity of the EOs against some postharvest phytopathogens (*Botrytis cinerea*, *Penicillium expansum*, *Aspergillus niger* and *Monilinia fructicola*), (iii) evaluate the in vitro antibacterial activity against *Bacillus megaterium*, *Clavibacter michiganensis*, *Xanthomonas campestris*, *Pseudomonas fluorescens* and *P. syringae* pv. *phaseolicola*, (iv) evaluate the effect of both studied EOs on the spore germination percentage and their minimum inhibitory concentration (MIC) against *M. fructicola*, and (v) study the possible phytotoxicity of the two EOs and their major constituents, carvacrol for *O. heracleoticum* and terpinen-4-ol for *O. majorana*, against tha germination and initial radicle growth of radish, lettuce, garden cress and tomato. The two EOs demonstrated promising in vitro antimicrobial and antifungal activities against all tested microorganisms. EOs showed high inhibition of spore germination percentage at the minimal inhibitory concentration of 500 and 2000 µg/mL, respectively. Moreover, both germination and radical elongation of selected plant species were sensitive to the oils.

## 1. Introduction

The control of pathogens and of weeds is a matter of importance in agriculture and synthetic pesticides are commonly used in their treatment, being highly effective in treatments in fields and stored products. However public concern is growing about the possible health and environmental risks related to the increased levels of pesticides and antibiotics in orchards and agriproducts. Considering also the resistance of pests to common agrochemicals, the need for alternative and safe pesticides and antibiotics encourages the exploitation of new sources, namely in the field of natural products [1]. In fact, there is a growing interest in a potential use of essential oils (EOs) as a replacement for traditional antibiotics, pesticides and herbicides. EOs are variable mixtures of volatile compounds produced by aromatic plants as secondary metabolites and are reported for their inhibitory activity against the growth of bacteria, yeasts and moulds, showing a variety of targets, particularly the membrane and cytoplasm, and in certain situations, they completely alter the morphology of the cells [2]. For a long time, EOs have been reported for their biological activities on seed germination and plant development [3].

Some essential oils (EOs) have been reported also for their allelopatic potential; in fact, they are capable of inhibiting the growth and/or the germination of competing plants in the surrounding environment [4], and they have also a broad spectrum of activity against plant pathogenic bacteria and fungi [5]. For these proprieties, there is a growing interest in the use of essential oils as an alternative for chemical herbicides, pesticides and antibiotics [6].

*Origanum heracleoticum* L. and *O. majorana* L. are two aromatic plants belonging to Labiatae family, native to the Mediterranean area.

*O. heracleoticum* L. is also known as ‘Greek oregano’; its EO, rich in phenols, shows antioxidant, antimicrobial, cytotoxic, antifungal proprieties. Moreover, it is used in aromatherapy as a co-adjuvant in the treatment of bronchitis, rheumatisms, gastroenteritis [7].

*O. majorana* L., usually known as a culinary additive, is also used in cosmetic industry and as a product with emotional, neurological and health benefits. Moreover, it is known for its antibacterial, antifungal and antiviral activities [8].

This study has been carried out to (i) investigate the chemical characterization of *O. heracleoticum* and *O. majorana*, (ii) evaluate the antimicrobial activity of the two EOs against some post-harvest phytopathogenic fungi: *Botrytis cinerea*, *Penicillium expansum*, *Aspergillus niger* and *Monilinia fructicola* and also some phytopathogenic bacteria (the Gram-positive *Bacillus megaterium* and *Clavibacter michiganensis* and the Gram-negative *Xanthomonas campestris, Pseudomonas fluorescens*, and *P. syringae* pv. *phaseolicola*), and (iii) study the possible phytotoxic activity of the two EOs and their main constituents on germination and radical elongation of *Raphanus sativus* L. (radish), *Lactuca sativa* L. (lettuce), *Lepidium sativum* L. (garden cress) and *Solanum lycopersicum* L. (tomato).

## 2. Results and Discussion

### 2.1. GC-MS Analysis

**Figure 1:**
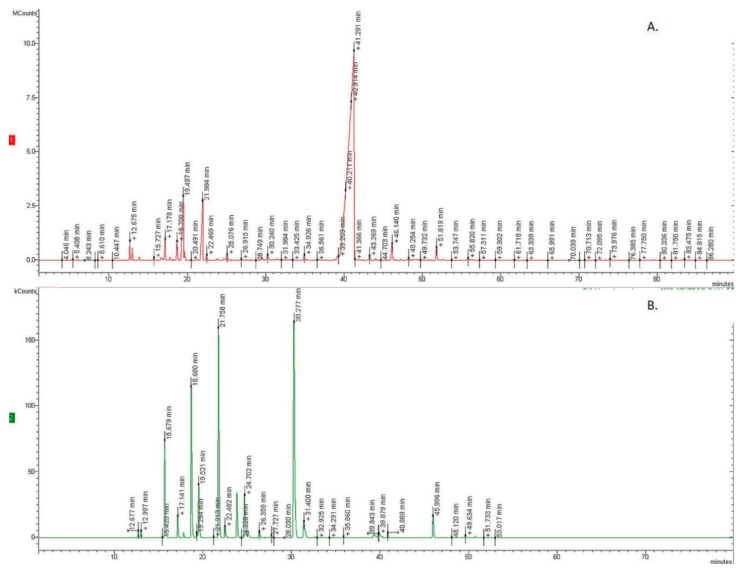
The Gas Chromatography (GC) profile of *O. heracleoticum* (**A**) and *O. majorana* essential oils (**B**).

Hydrodistillation of the aerial parts of *O. heracleoticum* and *O. majorana* furnished pale yellow oils in 1.8% and 0.4% yield on a dry mass basis, respectively. Table 1 and Table 2 show respectively the chemical composition of the two EOs in percent; compounds are listed according to their elution order on a HP-5MS column. The GC profile of two essential oils are present in Figure 1. Altogether, 55 compounds were identified, 35 for *O. heracleoticum*, accounting for 97.8% of the total oil, and 20 for *O. majorana*, accounting for 98.0% of the total oil.

Oxygenated monoterpenes (80.1%) are the main constituents in *O. heracleoticum* EO, and monoterpene hydrocarbons (54.0 %) are present in major percent in *O. majorana* EO.

In the oil from *O. heracleoticum* carvacrol (77.8%), *p*-cimene (5.3%), γ–terpinene (4.9%) and (E)-caryophyllene (1.3%) are the main components. In the oil from *O. majorana*, the main compounds are terpinen-4-ol (29.6%), δ-2-carene (20.1%), camphene (13.4%) and α-pinene (7.9%).

Other compounds, in a lesser amount are germacrene A (0.8%), α-thujene (0.7%) and terpinen-4-ol (0.5%) for *O. heracleoticum*, and *trans* isolimonene (5.4%), *cis*-sabinene hydrate (5.4%), α-terpinene (4.2%) and α-terpineol (2.8%) for *O. majorana*.

Our data about the chemical composition of *O. heracleoticum* EO agrees with data previously reported, which identified carvacrol as the main component of this oil. Conversely, in our study thymol, generally considered as one of principal constituents (ranging from 7.47–42.8% of total oil) was present in a very low quantity (0.3%) [9,10,11].

The chemical composition of our *O. majorana* EO is similar to data reported in literature. Terpinen-4-ol was confirmed as the most abundant component of *O. majorana* EO, as also demonstrated by Abbassy and co-workers, which reported a percentage of such compound ranging from 29.9% to 38.8% [12]. Similarly, γ-terpinene was reported as one of the major components [12,13]; in our study its isomer, α-terpinene, was present. Such a scenario did not coincide with data reported by Brosche et al. [13], which indicated *cis*-sabinene hydrate as one of main compounds of *O. majorana* EO present in equal percent to terpinen-4-ol; on the contrary, in our sample its concentration amounted to just 5.4%. Furthermore, in our analysis, we did not find 1,8-cineole, which conversely is indicated as the main component of *O. majorana* EO from Salerno (Italy) [3].

### 2.2. Antifungal In Vitro Test

**Figure 2:**
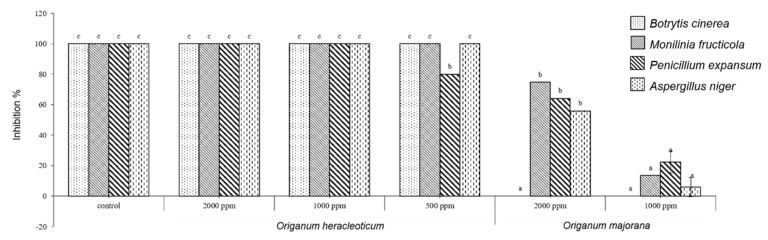
Antifungal activity of *O. heracleoticum* and *O. majorana* EOs. Bars with different letters for each tested fungi indicate mean values significantly different at *p* < 0.05 according to *Tukey* B test between different tested concentrations. Data are expressed as mean ± SDs.

*O. heracleoticum* EO was able to inhibit significantly the growth of all tested fungi and at all concentrations tested, instead *O. majorana* EO inhibited only three fungi (*M. fructicola*, *P. expansum* and *A. niger*) (Figure 2). Our results confirm the data of previous studies in which *O. heracleoticum* essential oil inhibited mycelial growth of *Penicillium expansum* isolated from organic cultivation of tomato [14]; and of *Monilinia* species [6].

Different studies reported that *O. majorana* essential oil showed antifungal activity against *B. cinerea* and *P. expansum* [1,15], whereas in our study this essential oil was not active against *B. cinerea.* No studies reported data on *O. heracleoticum* essential oil against *B. cinerea* and *A. niger* and on *O. majorana* essential oil against *A. niger.*

However, different studies reported an antifungal activity of plants belonging to *Origanum* genus. For example, *O. dictamnus* essential oil is active against the growth of *B. cinerea* [16]; *O. vulgare* against *P. expansum* [17,18] and *O. minutiflorum* against *A. niger* [19].

The efficacy of these EOs probably depends on their bioactive lipophilic constituents that play an essential role in degradation of fungal phytopathogens cell membrane [20]. Moreover, carvacrol, the main constituent of *O. heracleoticum* essential oil can act on the loss of proton gradient disturbing the cellular membrane [21].

### 2.3. Antibacterial In Vitro Test

**Figure 3:**
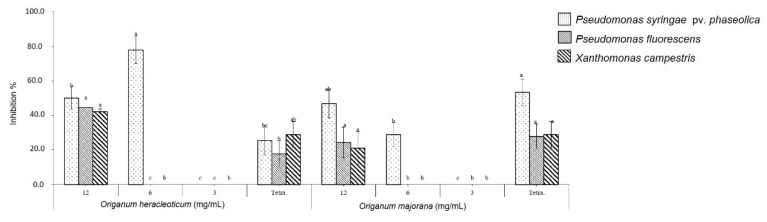
Antibacterial activity of *O. heracleoticum* and *O. majorana* EOs against G-ve bacteria. Bars with different letters for each tested bacteria indicate mean values significantly different at *p* < 0.05 according to the Tukey B test between different tested concentrations. Data are expressed as mean ± SDs.

**Figure 4:**
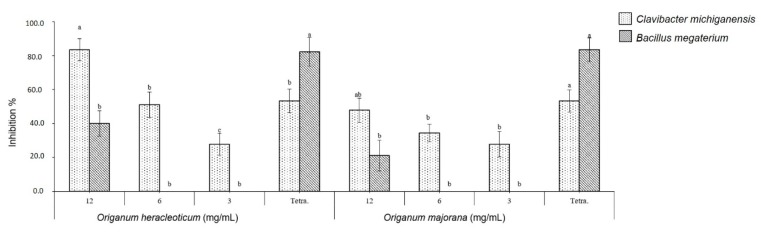
Antibacterial activity of *O. heracleoticum* and *O. majorana* EOs against Gram-positive bacteria. Bars with different letters for each tested bacteria indicate mean values significantly different at *p* < 0.05 according to the Tukey B test between different tested concentrations. Data are expressed as mean ± SDs.

The EOs showed a good antibacterial efficacy against the tested bacteria compared to the control. In fact, *O. heracleoticum* and *O. majorana* EOs were able to inhibit the growth of all Gram-negative bacteria tested at 12 mg/mL, whereas with concentration of 6 mg/mL the two EOs inhibited only the growth of *Pseudomonas syringae* pv. *phaseolicola* (Figure 3). Moreover, the two EOs showed more bactericidal effects against Gram-positive bacteria than against Gram-negative, inhibiting the growth of *Clavibacter michiganensis* at all tested concentrations in a dose-dependent manner (Figure 4) and *O. heracleoticum* was also active against *B. megaterium* but only at concentration of 12 mg/mL.

The potential efficacy of tested EOs against bacterial cells can depend on the ability of EOs to increase the cell membrane permeability and enhance the loss of ions, leakage of macromolecules and lysis [2].

Our results agree with previous studies on antibacterial activity of *O. heracleoticum* and *O. majorana* essential oil. In fact, they first showed an antibacterial activity against *Bacillus megaterium* [22], whereas *P. aeruginosa* appear to be the most resistant to the action of this essential oil [23]. Instead, *O. majorana* EO showed antibacterial activity against several plant pathogenic bacteria, such as *B. megaterium*, *Clavibacter michiganensis*, *Xanthomonas campestris*, and *P. syringae* pv. *Phaseolicola* [15,24,25]. However, no studies reported antibacterial activity of *O. heracleoticum* EO against *C. michiganensis*, *X. campestris* and *P. syringae* pv. *phaseolicola* and of *O. majorana* EO against *P. fluorescens*.

### 2.4. Fungal Spore Germination Assay

As presented in Table 3, *O. heracleoticum* and *O. majorana* EOs significantly inhibited the fungal spore germination of *M. fructicola* (*p* < 0.05) in a dose-dependent manner. In particular, the spore germination was reduced gradually from 79% to 94% and from 76% to 93% by increasing the concentration of *O. heracleoticum* and *O. majorana* EOs from 15 to 45 µg/µl, respectively. No previous studies reported data of spore germination assay for the two EOs against *M. fructicola.*

### 2.5. Determination of Minimum Inhibitory Concentration (MIC) (96-Well Microplate Method)

**Figure 5:**
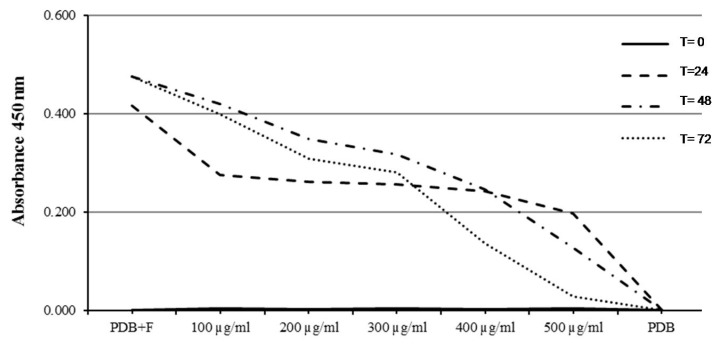
Minimum inhibitory concentration (MIC) of O. heracleoticum EO against *Monilinia fructicola*.

Results of the MIC assay of *O. heracleoticum* EO against *M. fructicola* were variable for the five different tested concentrations ranging between 100 and 500 µg/mL. The MIC value was recorded as the minimum absorbance reading close to the PDB value as a negative control. In particular, *O. heracleoticum* EO strongly inhibited the tested fungi and showed the lowest absorbance (0.028 nm) regarding the MIC value of the highest tested concentration (500 µg/mL) after 72 h of incubation at 24 ± 2 °C (Figure 5). It was observed also that there is a dramatic decrease of fungal mycelium growth by increasing the concentration of *O. heracleoticum* EO more than 300 µg/mL, especially after 48 and 72 h of incubation (Figure 5).

**Figure 6:**
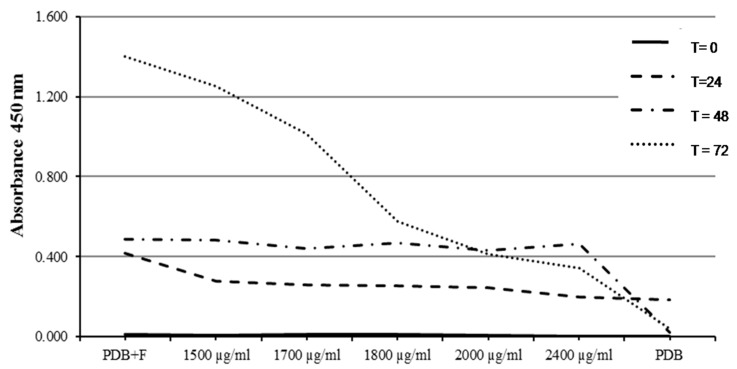
MIC of O. majorana EO against *Monilinia fructicola*.

On the other hand, the MIC of *O. majorana* EO against *M. fructicola* was less variable, especially for the first three days of incubation, whereas a high variability of fungal growth decrease was registered after 72 h for 2000 and 2400 µg/mL. The MIC value of *O. majorana* EO was 0.413 and 0.342 nm using 2000 and 2400 µg/mL, respectively (Figure 6). In addition, a decrease was observed with a concentration higher than 2000 µg/mL and after two days of incubation (Figure 6). There are no studies in literature that reported data of MIC assay for *M. fructicola* after treatment with *O. heracleoticum* and *O. majorana* essential oils.

### 2.6. Phytotoxic Activity

The two EOs and their main constituents, carvacrol and terpinen-4-ol, were evaluated for their activity against germination and radical elongation of radish, lettuce, garden cress and tomato.

**Figure 7:**
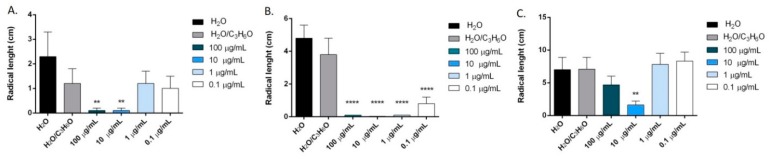
Phytotoxic activity of *O. heracleoticum* essential oil against radical elongation of *L. sativa* (**A**), *L. sativum* (**B**), *S. lycopersicum* (**C**). Results are expressed as the mean of three experiments standard deviation. ** *p* < 0.01, **** *p* < 0.0001 compared to control (ANOVA followed by Dunnett’s multiple comparison test).

**Figure 8:**
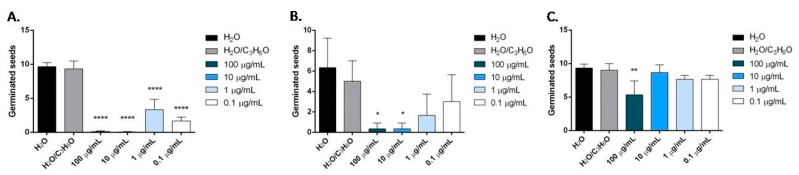
Phytotoxic activity of *O. heracleoticum* essential oil against germination of *L. sativa* (**A**), *L. sativum* (**B**), *S. lycopersicum* (**C**) seeds. Results are expressed as the mean of three experiments standard deviation. * *p* < 0.05, ** *p* < 0.01, **** *p* < 0.0001 compared to control (analysis of variance (ANOVA) followed by Dunnett’s multiple comparison test).

*O. heracleoticum* EO seemed to be effective against radical elongation and germination of *L. sativa*, *L. sativum* and *S. lycopersicum* (Figure 7 and Figure 8). In particular, the treatment of seeds with concentrations of 100 and 10 µg/mL of the oil inhibited the radical elongation of *L. sativa*; all doses tested affected significantly the radical elongation of *L. sativum* and only the concentration of 10 µg/mL inhibited the grown of *S. lycopersicum* (Figure 7). However, *O. heracleoticum* EO was active against its germination (Figure 8).

Our results agree with Elshafie et al. [22]. In fact, they demonstrated that the EO obtained from *O. heracleoticum* growing in Basilicata (Italy) inhibited germination and radical elongation of *L. sativum* and *R. sativus*. Moreover, our data confirmed the strong phytotoxic effect of this EO also tested on growth of *Arabidopsis* rosettes [26].

**Figure 9:**
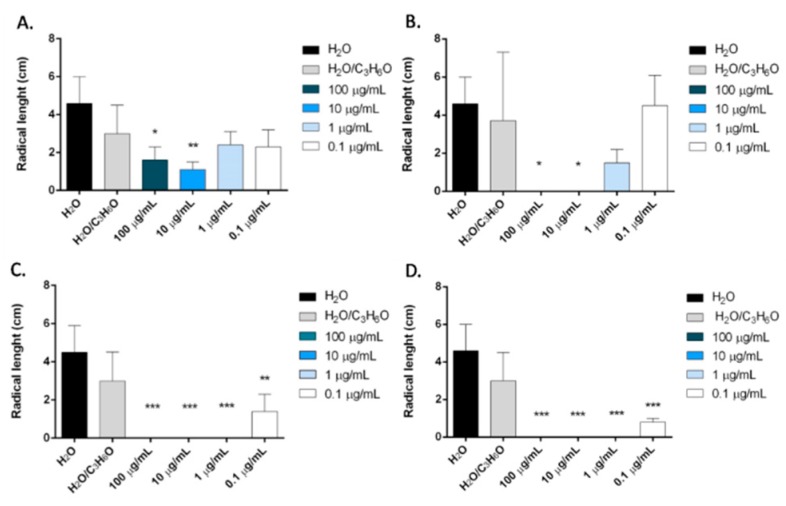
Phytotoxic activity of carvacrol against radical elongation of *R. sativum* (**A**), *S. lycopersicum* (**B)**, *L. sativa* (**C)**, *L. sativum* (**D**). Results are expressed as the mean of three experiments standard deviation. * *p* < 0.05, ** *p* < 0.01, *** *p* < 0.001 compared to control (ANOVA followed by Dunnett’s multiple comparison test).

**Figure 10:**
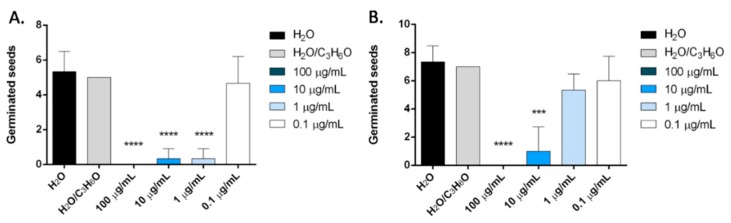
Phytotoxic activity of carvacrol against germination of *L. sativa* (**A**) and *S. lycopersicum* (**B**). Results are expressed as the mean of three experiments standard deviation. *** *p* < 0.001, **** *p* < 0.0001 compared to control (ANOVA followed by Dunnett’s multiple comparison test).

Carvacrol was more active than *O. heracleoticum* EO against radical elongation of all tested seeds and germination of *L. sativa* and *S. lycopersicum* (Figure 9 and Figure 10). In fact, this compound inhibited significantly, at all doses tested, the radical elongation of *L. sativa* and of *L. sativum* and at concentration of 100 µg/mL and of 10 µg/mL the radicle elongation of *R. sativum* and *S. lycopersicum* seeds (Figure 9*)*. Moreover, carvacrol inhibited the germination of *L. sativa*, and *S. lycopersicum*, at concentrations of 100, 10, 1 µg/mL and of 100 and 10 µg/mL, respectively (Figure 10).

Our data are in agreement with previous studies on the herbicidal effect of carvacrol. In fact, this compound inhibited the germination and seedling growth of *Amaranthus retroflexus*, *Chenopodium album* and *Rumex crispusa, Lolium rigidum,* *Lactuca sativa* and *Sorghum bicolour* [27,28,29], and can cause chromosomal alterations in the meristematic cells of *L. sativa* [30].

**Figure 11:**
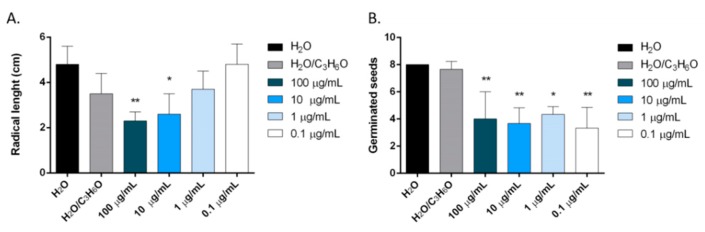
Phytotoxic activity of *O. majorana* essential oil against radical elongation of *L. sativum* (**A**) and against germination of *L. sativa* (**B**) seeds. Results are expressed as the mean of three experiments standard deviation. * *p* < 0.05, ** *p* < 0.01 compared to control (ANOVA followed by Dunnett’s multiple comparison test).

*O. majorana* EO inhibited the radical length of *Lepidium sativum* at concentrations of 100 and 10 µg/mL and germination of *Lactuca sativa* at all concentration tested (Figure 11).

There is only one previous study on phytotoxic activity of *O. majorana* EO [3]. In this research the EO was active on both radical elongation and germination of *L. sativa*, *L. sativum* and *R. sativum*, but this could be due to different chemical compositions of this EO, which was rich mainly of 1,8 cineole.

**Figure 12:**
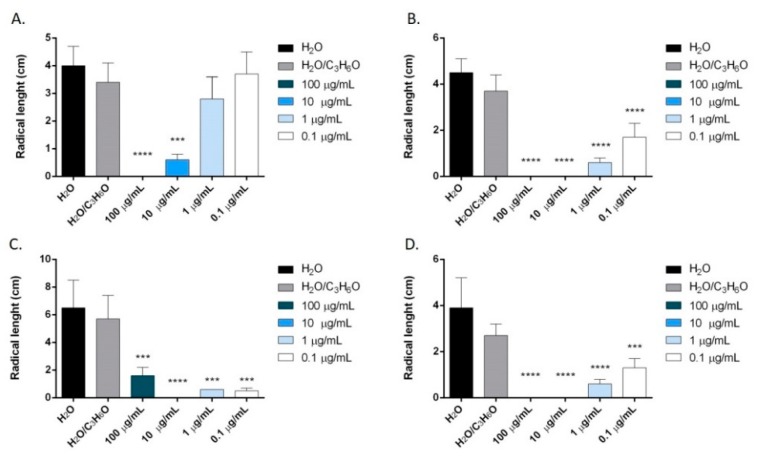
Phytotoxic activity of terpinen-4-ol against radical elongation of *L. sativa* (**A**), *L. sativum* (**B**), *S. lycopersicum* (**C**), *R. sativum* (**D**). Results are expressed as the mean of three experiments standard deviation. *** *p* < 0.001, **** *p* < 0.0001 compared to control (ANOVA followed by Dunnett’s multiple comparison test).

**Figure 13:**
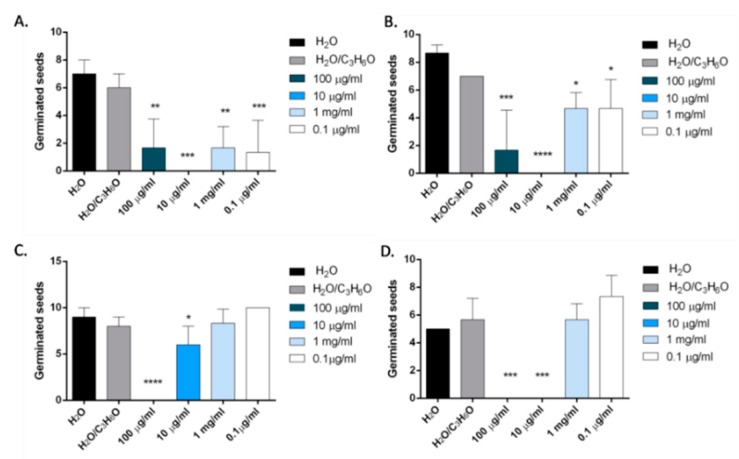
Phytotoxic activity of terpinen-4-ol against germination of *S. lycopersicum* (**A**), *R. sativum* (**B**), *L. sativa* (**C**), *L. sativum* L. (**D**). Results are expressed as the mean of three experiments standard deviation. * *p* < 0.05, ** *p* < 0.01, *** *p* < 0.001, **** *p* < 0.0001 compared to control (ANOVA followed by Dunnett’s multiple comparison test).

In our study, we also evaluated for the first time the potential phytotoxic activity of terpinen-4-ol, the main constituent of *O. majorana* EO. Results showed that this compound inhibited the radical elongation and germination of all tested seeds (Figure 12 and Figure 13).

In particular, on *L. sativum, S. lycopersicum* and *R. sativum* seeds, all concentrations tested significantly reduced the radical length, instead, on *L. sativa* seeds, the effect was evident at concentrations of 100 and 10 µg/mL (Figure 12).

The inhibition of germination was more evident on *Solanum lycopersicum* seeds, where an inhibition of germination was observed for all concentrations used (100–0.1 μg/mL). The effectiveness was less evident on *R. sativum*, *L. sativa* and *L. sativum* seeds, and only the two major concentrations used (100 and 10 μg/mL) demonstrated dose-dependent inhibitory activity (Figure 13).

## 3. Materials and Methods

### 3.1. Plant Materials

Representative homogeneous samples of *O. heracleoticum* and *O. majorana* were collected in September 2018, in Montecorice, Salerno province (Southern Italy). The plants were identified by Prof. V. De Feo, based on Flora d’Italia [31] and a voucher specimen of each plant has been deposited in the herbarium of the Medical Botany Chair of University of Salerno.

### 3.2. Isolation of the Volatile Oil

One kilogram of dried aerial parts of *O. heracleoticum* and of *O. majorana* were ground in a Waring blender and then subjected to hydrodistillation for 3 h according to the standard procedure described in the European Pharmacopoeia [32]. The oils were solubilized in *n*-hexane, filtered over anhydrous sodium sulphate and stored under N_2_ at +4 °C in the dark until tested and analyzed.

### 3.3. Gas Chromatography-Flame Ionization Detector (GC-FID) Analysis

Analytical gas chromatography was carried out on a Perkin–Elmer Sigma-115 gas chromatograph equipped with a FID and a data handling processor. The separation was achieved using a HP-5 MS fused-silica capillary column (30 m × 0.25 mm i.d., 0.25 µm film thickness). Column temperature: 40 °C, with 5 min initial hold, and then to 270 °C at 2 °C/min, 270 °C (20 min); injection mode splitless (1 µL of a 1:1000 *n*-hexane solution). Injector and detector temperatures were 250 °C and 290 °C, respectively. Analysis was also run by using a fused silica HP Innowax polyethylenglycol capillary column (50 m × 0.20 mm i.d., 0.25 µm film thickness) (Santa Clara, CA, USA). In both cases, helium was used as the carrier gas (1.0 mL/min).

### 3.4. GC/Mass Spectroscopy (MS) Analysis and Identification of Constituents

Analyses were performed on an Agilent 6850 Ser. II apparatus (Santa Clara, CA, USA), fitted with a fused silica DB-5 capillary column (30 m × 0.25 mm i.d., 0.33 µm film thickness) (Santa Clara, CA, USA), coupled to an Agilent Mass Selective Detector MSD 5973; ionization energy voltage 70 eV; electron multiplier voltage energy 2000 V.

Mass spectra were scanned in the range 40–500 amu, scan time 5 scans/s. Gas chromatographic conditions were as reported above, and the transfer line temperature was 295 °C.

Most constituents were identified by gas chromatography by comparison of their Kovats retention indices (Ri) (determined relative to the tR of *n*-alkanes (C_10_–C_35_)), with either those of the literature [33,34,35,36] and mass spectra on both columns with those of authentic compounds available in our laboratories by means of the National Institute of Standards and Technology (NIST) and Wiley 275 libraries [37]. The relative concentrations of the single components were obtained by peak area normalization.

### 3.5. Antifungal Activity

#### 3.5.1. Fungal Isolates

The tested fungi *Botrytis cinerea*, *Penicillium expansum*, *Aspergillus niger* and *Monilinia fructicola* were maintained as pure cultures at 4 °C in the mycotheca of the School of Agricultural, Forestry, Food and Environmental Sciences (SAFE), Basilicata University, Potenza, (Italy) and activated on potato dextrose agar (PDA) at 24 ± 2 °C. Identification of the isolates was carried out by morphological and molecular methods based on polymerase chain reaction (PCR). The total nucleic acids were extracted from pure culture with a commercial kit (Dneasy Plant mini kit, Qiagen, Hilden, Germany) according to the manufacturer’s instructions. The DNA was amplified using the universal primer pair ITS4/ITS5 [38]. The amplicon obtained were directly sequenced and the resulting sequences were compared with those available in GenBank using Basic Local Alignment Search Tool software (BLAST-USA) [39]. Gene sequencing of the internal transcribed spacer (ITS) region confirmed the identification done by traditional methods.

#### 3.5.2. In Vitro Fungicidal Activity

The antifungal activity of *O. heracleoticum* and *O. majorana* were assessed at 2000, 1000 and 500 ppm incorporating directly the EO in potato dextrose agar (PDA) with 0.2% Tween 20 following the method reported by Elshafie et al. [40]. Fungal discs of 0.5 cm from fresh cultures were inoculated in the center of Petri dishes containing 14 mL of PDA. All tested Petri dishes were incubated at 24 ± 2 °C for 96 h and later the diameter of fungal mycelium growth was measured in mm. PDA plates incorporated only with Tween 20 (0.2%) were inoculated with the same tested fungi and used as negative control. The experiment was carried out in triplicate and the fungitoxic effect was expressed as a percentage of growth inhibition (PGI) according to the formula of Zygadlo et al. [41], compared to azoxystrobin (80 µL.100 mL^−1^) as the positive control:

PGI (%) = 100 × (GC − GT) / GC,

where GC = the average diameter of fungus colony grown on PDA (control), and GT = the average diameter of fungus colony grown on PDA containing each EO.

### 3.6. Antibacterial Activity Assay

#### 3.6.1. Bacterial Isolates

The test was performed using different Gram-positive (*Bacillus megaterium* and *Clavibacter michiganensis)* and Gram-negative (*Xanthomonas campestris, Pseudomonas fluorescens*, and *P. syringae* pv. *phaseolicola)* bacteria. The tested strains were cultured on King B (KB) media and incubated at 30 ± 2 °C for 72 h. All tested bacteria were previously identified by molecular method (PCR). All bacterial strains were maintained as lyophils at −20 °C in the collection present at SAFE and sub-cultured on King B media (KB) for 48 h at 25 °C.

#### 3.6.2. Antibacterial Activity Test

The antibacterial activity was assessed following the method reported by Elshafie et al. [42]. A suspension of each tested bacteria was prepared at 10^8^ CFU mL^−1^ from fresh culture (24 h) adjusted by spectrophotometer UNICAM-UV 500 (Cambridge, UK). A mixture of 3 mL soft agar (0.7 %) and 1 mL of bacterial suspension 10^8^ CFU mL^−1^ was prepared and poured into a KB plate (10 mL). 10 µL of each EO concentration at 12, 6, 3 mg/mL and tetracycline at 1600 µg mL^−1^ were applied over blank discs (6 mm, OXOID).

The antibacterial activity was evaluated by measuring the diameter of the inhibition zone (mm) around each treatment compared to tetracycline (positive control). The bacterial inhibition percentage (BIP) was calculated following the equation proposed by Elshafie et al. [43].

BIP (%) =100 − [(GC − GT)/GC × 100],

where: BIP represents the bacterial inhibition percentage, GC the average diameter of bacterial grown in control plate in mm and GT the average diameter of inhibition zone in mm.

### 3.7. Fungal Spore Germination Assay

The inhibition of fungal spore germination of the most inhibited fungi resulting from the previous assay was determined using the liquid dilution method with three different concentrations of EO (15, 22 and 45 µg/µL). An aliquot of the three solutions was applied in contact with the tested fungi (10^8^ spores/mL) for 6 h at 28 °C and 80% relative humidity. Only PDB has been used as negative control (−ve). Fifty μL of suspension containing spores and of solutions containing different EO concentration were dropped onto a Thoma cell counting chamber and the percentage spore germination was recorded after 36 h using a light microscope compared to negative control.

### 3.8. Determination of MIC (96-Well Microplate Method)

The minimum inhibitory concentration (MIC) was carried out against the most inhibited pathogenic fungi using a *96*-well microplate (Nunc MaxiSorp^®^, Vedbaek, Denmark) by a micro-dilution method following the method of Elshafie et al. [44]. Four milliliters of a liquid suspension from fresh fungal cultures (96 h) was prepared at 10^8^ spore/mL using Turbidimetry at optical density, OD ≈ 0.2 nm. The EOs were prepared in potato dextrose broth (PDB) at 1500, 1700, 1800, 2000 and 2400 ppm for *O. majorana* EO and 500, 400, 300, 200 and 100 ppm for *O. heracleoticum* EO. The incorporation has been carried out using the mixture between PDB, Tween 20 (0.2%) and the above-mentioned concentrations of tested EOs. The above-mentioned concentrations were proposed considering the results obtained from the initial antifungal screening assay. Two hundred microliters of each prepared concentration and 100 µL of the prepared fungal suspension were added in each well; then the microplate was incubated at 24 ± 2 °C and the absorbance was read at λ = 450 nm using the ELISA microplate reader instrument (DAS s.r.l., Rome, Italy) after 24, 48, and 72 h. The experiment was replicated in triplicate.

### 3.9. Phytotoxic Activity

The phytotoxic activity was evaluated considering the effect on germination and radical elongation of four species: *Raphanus sativus*, *Lactuca sativa*, *Lepidium sativum* and *Solanum lycopersicum*. These seeds are usually used in phytotoxicity assays for their easy germinability. All tested seeds were purchased from Blumen group srl (Emilia Romagna, Italy). The seeds were surface-sterilized in 95% ethanol for 15 s and sown in Petri dishes (Ø = 90 mm), containing three layers of Whatman filter paper, impregnated with distilled water (7 mL, control) or the tested solution of the EO (7 mL), at the different doses. The germination assay was carried out at 20 ± 1 °C, with natural photoperiod. The EOs, in water–acetone mixture (99.5:0.5), were assayed at the doses of 100, 10, 1 and 0.1 µg/mL. Controls performed with the water–acetone mixture alone showed no appreciable differences in comparison to controls in water alone. Seed germination was observed directly in Petri dishes every 24 h. A seed was considered germinated when the protrusion of the radicle became evident [45]. After 120 h (on the fifth day), the effects on radicle elongation were measured in cm. Each determination was repeated three times, using Petri dishes containing 10 seeds each. Data are expressed as the mean ± SD for both germination and radicle elongation.

### 3.10. Statistical Analysis

Results of the antimicrobial activity were statistically analyzed using the Statistical Package for the Social Sciences (SPSS) (version 13.0, Prentice Hall: Chicago, IL, USA, 2004) and applying one way ANOVA and Tukey post hoc test for detecting any significant differences between the different concentrations of tested EOs at *p* < 0.05. Instead, data of each experiment for determination of phytotoxicity were statistically analyzed using the GraphPad Prism 6.0 software (GraphPad Software Inc., San Diego, CA, USA), followed by comparison of means (one-way ANOVA) using Dunnett’s multiple comparisons test, at the significance level of *p* < 0.05.

## 4. Conclusions

Our results showed that the two EOs could be utilized to control plant infections of all the tested fungi and Gram-positive and Gram-negative bacteria. Moreover, they are can inhibit the germination and the radical elongation of the tested species to different extents. The possible application of EOs in these fields is very promising because they are compatible with both environmental and human health. However, further investigations will need to study the antifungal and antibacterial efficacy of the single components of *O. heracleoticum* and *O. majorana* essential oil, and to evaluate their possible phytotoxic effects on crop species to confirm their possible use on a large scale as pesticides and/or herbicides.

## Figures and Tables

**Table 1:** Chemical composition of *O. heracleoticum* essential oil.

No.	Compounds	%	KI ^a^	KI ^b^	Identification ^c^
**1**	α-Thujene	0.7	856	930	1,2,3
**2**	α-Pinene	0.4	860	939	1,2,3
**3**	Camphene	0.1	871	954	1,2,3
**4**	Sabinene	0.4	896	975	1,2,3
**5**	β-Pinene	1.0	914	979	1,2,3
**6**	α-Phellandrene	0.2	922	1002	1,2,3
**7**	δ-3-Carene	0.1	927	1011	1,2
**8**	α-Terpinene	1.1	934	1017	1,2,3
**9**	ρ–Cimene	5.3	944	1024	1,2
**10**	Limonene	0.3	946	1029	1,2,3
**11**	1,8 Cineol	0.1	947	1031	1,2,3
**12**	(E)-β-Ocimene	0.1	966	1050	1,2,3
**13**	γ-Terpinene	4.9	977	1059	1,2,3
**14**	*cis*-Sabinene hydrate	0.3	983	1070	1,2
**15**	Terpinolene	0.1	1000	1088	1,2
**16**	Linalool	0.4	1017	1096	1,2,3
**17**	*trans*–Sabinene hydrate	t	1033	1098	1,2
**18**	*allo*-Ocimene	0.1	1040	1132	1,2
**19**	Borneol	0.4	1073	1169	1,2,3
**20**	Terpinen-4-ol	0.5	1083	1177	1,2,3
**21**	α-Terpineol	0.1	1097	1188	1,2,3
**22**	*cis*–Dihydrocarvone	0.1	1110	1192	1,2
**23**	*trans*-Dihydrocarvone	t	1106	1200	1,2
**24**	Thymol methyl ether	0.3	1146	1235	1,2
**25**	Thymoquinone	t	1151	1252	1,2
**26**	Thymol	0.1	1204	1290	1,2,3
**27**	Carvacrol	77.8	1218	1299	1,2,3
**28**	Eugenol	0.2	1261	1359	1,2,3
**29**	(E)-Caryophyllene	1.3	1303	1419	1,2
**30**	Aromadendrene	t	1320	1441	1,2,3
**31**	α-Humulene	0.2	1335	1454	1,2,3
**32**	Aceto-vanillone	0.1	1346	1482	1,2
**33**	Germacrene A	0.8	1389	1509	1,2
**34**	Caryophyllene oxide	0.2	1452	1583	1,2,3
**35**	α Bisabolol	t	1150	1685	1,2,3
	Total	97.8			
	Monoterpene hydrocarbons	15.1			
	Oxygenated monoterpenes	80.1			
	Sesquiterpene hydrocarbons	2.3			
	Oxygenated sesquiterpenes	0.2			

^a^ Linear retention index on a HP-5MS column; ^b^ Linear retention index on a HP Innowax column; ^c^ Identification method: 1 = linear retention index; 2 = identification based on the comparison of mass spectra; 3 = Co-injection with standard compounds.

**Table 2:** Chemical composition of *O. majorana* essential oil.

No.	Compounds	%	K ^a^	K ^b^	Identification ^c^
**1**	α-Thujene	0.4	856	930	1,2,3
**2**	α-Pinene	7.9	895	939	1,2,3
**3**	α– Fenchene	2.2	914	951	1,2
**4**	Camphene	13.4	934	954	1,2,3
**5**	Verbetene	0.4	942	967	1,2
**6**	*trans-*Isolimonene	5.4	945	984	1,2
**7**	δ-2-Carene	20.1	974	1002	1,2
**8**	α-Terpinene	4.2	1000	1017	1,2,3
**9**	*cis*- Sabinene hydrate	5.4	1009	1070	1,2
**10**	Linalool	1.0	1017	1096	1,2,3
**11**	*cis*-*p*-Menth-2-en-1-ol	0.8	1033	1121	1,2
**12**	*allo*-Ocimene	t	1040	1132	1,2,3
**13**	*trans*-*p*-Menth-2-en-1-ol	0.4	1051	1177	1,2
**14**	Terpinen-4-ol	29.6	1083	1177	1,2
**15**	α-Terpineol	2.8	1087	1188	1,2
**16**	Thymol	0.3	1204	1290	1,2,3
**17**	Carvacrol	0.6	1218	1299	1,2,3
**18**	δ-Elemene	0.5	1227	1338	1,2
**19**	(E)-Caryophyllene	2.2	1003	1419	1,2,3
**20**	*cis*-Muurola-3,5-diene	0.2	1353	1450	1,2
	Total	98.0			
	Monoterpene hydrocarbons	54.0			
	Oxygenated monoterpenes	40.9			
	Sesquiterpene hydrocarbons	2.9			

^a^ Linear retention index on a HP-5MS column; ^b^ Linear retention index on a HP Innowax column; ^c^ Identification method: 1 = linear retention index; 2 = identification based on the comparison of mass spectra; 3 = Co-injection with standard compounds.

**Table 3:** Evaluation the effect of *O. heracleoticum* and *O. majorana* EOs on the spore germination of *Monilinia fructicola.*

EOs		^*^ Spore/mL	^**^ Spore Germination %	^***^ Inhibition Rate %
	Control	80.0 × 10^4^	100.0	0.0
*O. heracleoticum* EO	15 µg/µL	16.7 × 10^4^	20.9 ^a^	79.1 ^ab^
	22 µg/µL	92.5 × 10^4^	11.6 ^b^	88.4 ^a^
	45 µg/µL	45.0 × 10^4^	5.6 ^b^	94.4 ^a^
*O. majorana* EO	15 µg/µL	16.8 × 10^4^	23.5 ^a^	76.5 ^ab^
	22 µg/µL	10.2 × 10^4^	12.8 ^ab^	87.2 ^a^
	45 µg/µL	5.5 × 10^4^	6.9 ^ab^	93.1 ^a^

(*): Spores/mL = average number of spores ×10^4^; (**): Spore germination % = (total number of spore in treated media / total number of spores in control ×100; (***): Inhibition rate % = percentage of spore germination in control—percentage of spore germination in treated media, and (a, b, ab) different letters indicates the significant differences at *p* ≤ 0.05.
